# A maturity-aware decision support system for smart circular cities: integrating ISO 37122 and ISO 59020 for policy-oriented assessment

**DOI:** 10.1016/j.mex.2026.104088

**Published:** 2026-08-03

**Authors:** Novi Dian Nathasia, Widowati, Aris Puji Widodo

**Affiliations:** a Doctoral Program of Information System Postgraduate School, Universitas Diponegoro, Indonesia; b Department of Mathematics, Faculty of Science and Mathematics, Universitas Diponegoro, Indonesia; c Department of Information System, Faculty of Communication Technology and Informatics, Universitas Nasional, Indonesia

**Keywords:** Smart circular city, Decision support, ISO 37122, ISO 59020, Circularity

## Abstract

Municipal scorecards frequently compress heterogeneous smart city and circular economy evidence into composite values that remain difficult to audit, compare, or translate into actionable policy. This article introduces MA-SCC-DSS, a reproducible, maturity-aware decision-support method grounded in Design Science Research artifact logic and informed by ISO 37122 for smart city assessment and ISO 59020 for circularity evaluation. Its methodological contribution is not a new standard or maturity scale, but the executable coupling of standards-informed mapping, evidence-confidence tagging, component-level rule gates, and tiered circularity activation within a traceable score-to-action pathway. The method transforms annual component scores and associated metadata into validated input tables, standards-informed indicator mappings, confidence tags, maturity bands, diagnostic alerts, and a policy-facing roadmap. MA-SCC-DSS was deliberately designed for settings where circularity data are uneven or incomplete, enabling practitioners to begin with aggregated scorecards and progressively incorporate sectoral indicators as richer data become available. The present validation establishes implementation correctness and internal consistency for one three-year illustrative dataset; it does not establish cross-municipality validity.

## Graphical abstract

**Figure:**
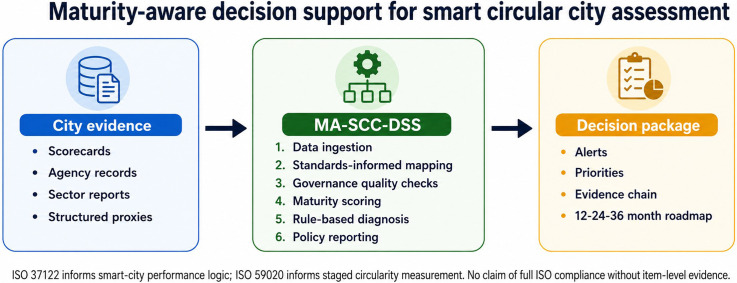

**Key points**

An executable analytical bridge that couples ISO 37,122- and ISO 59,020-informed mapping, evidence-confidence tagging, maturity rules, and tiered circularity activation in a traceable six-layer score-to-action pipeline;

A transparent and auditable pathway from raw component scores to maturity classifications, rule-triggered diagnostics, and prioritised roadmap actions; and

A fully reproducible demonstration, comprising illustrative data and Python code, enabling independent verification of classifications, diagnostic alerts, and sensitivity outcomes.

Alt-text: Unlabelled box dummy alt text

## Specifications table

**Table:** 

Subject area	Decision Sciences; Environmental Science; Computer Science; Social Sciences
More specific subject area	Urban analytics, smart city assessment, circular economy measurement, public-sector decision support, and urban data governance
Name of your method	Maturity-Aware Smart Circular City Decision Support System (MA-SCC-DSS)
Name and reference of original method	No single prior method is reproduced. MA-SCC-DSS adapts and operationally couples the following established foundations: (i) Design Science Research artifact-building and evaluation logic (Hevner AR, March ST, Park J, Ram S. Design science in information systems research); (ii) maturity-aware assessment for smart/circular cities and public organizations (Damianou A, Vayona A, Demetriou G, Katos V. An actionable maturity planning model for smart, circular cities); (iii) ISO 37,122 smart-city indicator logic (International Organization for Standardization. ISO 37,122:2019 Sustainable cities and communities — Indicators for smart cities); and (iv) ISO 59,020 circularity measurement logic (International Organization for Standardization. ISO 59,020:2024 Circular economy — Measuring and assessing circularity performance).
Resource availability	Demonstration data and reproducibility code should be submitted as supplementary files and deposited in mendeley data. https://data.mendeley.com/preview/649dn72968?a = 00bf880d–8538–4470-b757–913124f952a1

## Background

Urban governments have long relied on dashboards, annual scorecards, and performance portals to track progress on smart-city and sustainability agendas [[1], [2], [3]]. While these tools bring welcome transparency to complex governance processes, they tend to reduce heterogeneous evidence drawn from multiple agencies, sectors, and reporting cycles into a single composite score [[4], [5], [6]]. This compression, though convenient, can mask deteriorating performance in specific domains, conceal weaknesses in underlying data quality, and make it harder for decision-makers to identify where action is most urgently needed [[7], [8], [9]].

These challenges are compounded when circular economy objectives are layered onto smart-city assessment frameworks [[10], [11], [12]]. Measuring circularity demands evidence spanning materials, water, energy, waste streams, reuse practices, recovery rates, and cross-sector resource flows — domains where municipal data tend to be less mature, less consistently collected, and less comparable than conventional service-delivery indicators [[13], [14], [15]]. Any method that requires comprehensive resource-flow accounting as a precondition is therefore likely to remain inaccessible in cities where data readiness varies considerably across departments and sectors [[16], [17], [18]].

MA-SCC-DSS was conceived in response to this gap, positioned as a decision-support method rather than a compliance instrument. It draws on ISO 37,122 to guide the interpretation of smart-city performance and on ISO 59,020 to structure the logic of circularity measurement [1,2]. Importantly, the method does not assert ISO certification or full conformance unless individual indicators have been explicitly mapped to the relevant standard requirements and supported by documented evidence. The standards serve instead as disciplined references for harmonizing indicators, setting measurement boundaries, interpreting outputs, and enabling circularity functions to be activated progressively as data availability improves.

The method was developed within a Design Science Research framework, reflecting the nature of its primary contribution: an operational artifact. Specifically, it provides a reusable pipeline capable of registering evidence, harmonizing inputs across sources, grading data confidence, assigning performance maturity bands, applying explicit diagnostic rules, and assembling a structured decision package suitable for policy audiences [[19], [20], [21], [22]]. This article presents the method in sufficient procedural detail to allow researchers, analysts, or municipal practitioners to reproduce it independently, using an annual scorecard dataset together with the accompanying analytical script.

## Methodological novelty and adaptation boundary

MA-SCC-DSS does not claim that ISO 37,122, ISO 59,020, maturity models, rule-based diagnosis, or Design Science Research are individually new. Its methodological novelty lies in their operational coupling into one executable transformation protocol with five linked features: (i) a minimum viable entry point using aggregated scorecards; (ii) dual standards-informed mapping with an explicit non-conformance boundary; (iii) confidence tags carried with each score into interpretation; (iv) component-level diagnostic gates that preserve a traceable score-to-band-to-alert-to-action path; and (v) tiered activation of circularity metrics as data maturity improves. Existing approaches generally address these elements separately [1,2,8,19,22]. The reusable contribution is therefore a governance-ready, auditable workflow that remains interpretable under heterogeneous data maturity, rather than a new ISO standard, universal maturity scale, or causal model.

The adaptation boundary is explicit: the ISO standards discipline indicator definitions and measurement boundaries; the maturity cut points serve as configurable operational defaults; and the diagnostic gates are transparent heuristics subject to local calibration. The artifact contribution is the orchestration of these elements, the evidence-quality safeguards, and the preserved path from input evidence to policy recommendation.

## Method details

### Overview of the workflow

MA-SCC-DSS translates annual urban performance evidence into decision outputs that are both maturity-aware and oriented toward practical policy use. The method was designed with municipal scorecards, smart-city assessments, circular economy readiness evaluations, and broader public-sector monitoring systems in mind. It proves most valuable in contexts where analysts possess richer information than a single composite score can convey, yet fall short of the fully disaggregated, real-time circularity data that more advanced platforms require.

At its core, the method accepts aggregated component scores as the minimum viable input, providing a workable entry point even where data infrastructure remains limited. From this baseline, analysts can progressively enrich the analysis by incorporating metadata, confidence flags, sectoral circularity indicators, API-connected data sources, or sensor-supported evidence as local data maturity develops over time. The resulting output makes no claim to causal explanation. Rather, it functions as an auditable first-pass diagnostic package — one that helps users locate maturity bottlenecks within the assessment and translate those findings into a set of prioritized, actionable policy responses, (Table 1).

**Table 1:** Overview of the MA-SCC-DSS pipeline.

Stage	Main task	Output
1. Data ingestion	Register scorecard values, reporting periods, sources, and institutional owners.	Structured input table
2. Harmonization and standards mapping	Normalize component labels and interpret available evidence through ISO 37,122-informed and ISO 59,020-informed logic.	Mapping notes and data dictionary
3. Governance quality control	Tag confidence, provenance, completeness, and definitional stability.	Confidence flags and quality notes
4. Scoring and maturity mapping	Retain official composites, calculate trends, compute sensitivity means, and assign maturity bands.	Component and composite maturity profile
5. Rule-based diagnosis	Apply explicit diagnostic gates to observed score patterns.	Triggered alerts and diagnostic meanings
6. Policy-facing reporting	Rank alerts and translate them into staged governance and measurement actions.	Executive scorecard, alert panel, priorities, and roadmap

### Required inputs and minimum data schema

The minimum implementation requires one row per assessment period and one column for each component score. The demonstration uses five components: Baseline, Output, Outcome, Impact, and Quick Win. These labels can be renamed if the local scorecard uses different terms, but the analyst must document the mapping before running the rule engine.

Required materials: (i) a CSV or spreadsheet containing annual component scores; (ii) definitions of the score scale; (iii) metadata on source, owner, and reporting period when available; (iv) the reproducibility script or equivalent implementation of the formulas and rules below; (v) a reporting template for documenting outputs; and (vi) a versioned configuration record documenting score transformations, maturity cut points, rule parameters, and the approval date, (Table 2).

**Table 2:** Minimum input schema.

Field	Required	Role in the method	Example
year	Yes	Defines the reporting period and enables trend analysis.	2022
baseline	Yes	Represents foundational readiness, governance capacity, enabling conditions, and data stewardship.	2.85
output	Yes	Represents delivered activities, services, reporting outputs, or implementation consistency.	2.07
outcome	Yes	Represents intermediate changes that appear after outputs are delivered.	2.92
impact	Yes	Represents durable effects and the credibility of the program-to-impact chain.	2.75
quick_win	Yes	Represents short-term gains that may or may not be institutionalized.	3.48
reported_composite	Recommended	Preserves the official scorecard value when it differs from the arithmetic mean of visible components.	2.83
metadata/confidence	Optional	Documents source, owner, proxy use, completeness, and definitional stability.	moderate confidence
sectoral_circularity_indicator	Optional	Allows the ISO 59,020-informed circularity layer to become more detailed when data improve.	waste recovery rate

Step 1 **Preparing and registering the scorecard evidence**

The process begins by assembling the raw materials of the assessment: annual scorecard values, component labels, reporting periods, institutional owners, and any source documentation that can be retrieved. These inputs are organized into a controlled table structured with one row per year. Column names are standardized across entries, and the provenance of each value is recorded explicitly. Analysts should further distinguish whether each value was directly measured, derived through calculation, or estimated by proxy, and should retain any official composite score that the city already publishes through its own reporting channels.

Before proceeding, several quality checks are essential. These include verifying the completeness of the dataset, identifying duplicate reporting periods, resolving inconsistent labeling across components, detecting values that fall outside the intended measurement scale, and clarifying any ambiguity in source ownership. Critically, imperfect evidence should not be discarded at this stage. Instead, problematic entries are flagged for transparency, ensuring that downstream recommendations do not carry unwarranted confidence. The output of this step is a validated input table ready to enter the harmonization process.

Step 2 **Harmonizing components and mapping them to standards-informed logic**

With a validated scorecard table and accompanying component definitions in hand, the second step involves aligning component names and definitions with the method's decision-support containers. The smart-city dimension is interpreted through performance logic informed by ISO 37,122, while the circularity dimension is structured according to measurement logic drawn from ISO 59,020. It is important to emphasize that this mapping is interpretive in nature and does not constitute a claim of certification or formal conformance with either standard. Cities working with aggregated data at the outset are not excluded from the process; item-level or sector-level indicators can be incorporated at a later stage as data availability and institutional capacity improve. The output of this step is a data dictionary and standards-informed mapping note., (Table 3).

**Table 3:** Standards-informed interpretation of the scorecard containers.

Decision-support container	Smart city interpretation	Circularity interpretation when data are available
Baseline	Readiness, governance capacity, data stewardship, interoperability, and enabling infrastructure.	System boundary definition, circularity data readiness, and ownership of resource-flow records.
Output	Operational delivery, service reporting, platform outputs, and implementation consistency.	Measured circular actions such as waste collection, water reuse programs, energy efficiency actions, or material recovery initiatives.
Outcome	Intermediate service or governance improvements resulting from outputs.	Changes in resource-use patterns, recovery rates, reuse adoption, or cross-sector coordination.
Impact	Longer-term public value, institutionalized performance, and durable effects.	Sustained circularity performance, environmental benefit evidence, and reduced dependence on linear resource use.
Quick Win	Short-term gains that create momentum but still need institutionalization.	Pilot circularity initiatives or early sectoral wins that require SOPs, budget anchors, and ownership to scale.

Step 3 **Adding governance quality and confidence tags**

Once components have been mapped, each is assigned a confidence tag derived from the available metadata. This tag characterizes the credibility of the underlying evidence base — capturing dimensions such as provenance, completeness, and definitional stability — and accompanies the score throughout the remainder of the analysis into the final recommendation package. The intent is to ensure that the strength of any subsequent recommendation remains proportionate to the quality of the evidence supporting it.

Step 4 **Calculating scores, trends, and maturity bands**

This step draws on the harmonized scorecard table, together with the official composite score where one has been published. When an official composite exists, it is retained as P_t. Annual change is then expressed as ΔP_t = P_t − P_(t − 1), capturing directional movement across reporting periods. As a sensitivity check, an arithmetic mean of visible components is computed as Mean_t = (Baseline_t + Output_t + Outcome_t + Impact_t + QuickWin_t) / 5, allowing analysts to observe whether the official composite diverges meaningfully from a simple average of its parts. Maturity bands are subsequently assigned to both component-level and composite-level values, yielding a structured maturity profile that carries forward into the diagnostic phase.

### Contextual calibration protocol

The cut points in Table 4 are transparent defaults for a 1–5 demonstration scale and are not universal municipal thresholds. Before implementation in another context, analysts should (i) verify score direction and range and, when necessary, transform values to a common scale; (ii) anchor cut points to an adopted local rubric, policy target, or empirical distribution; (iii) document the rationale and responsible approving stakeholders; (iv) freeze and version the configuration before analysis; and (v) repeat the sensitivity analysis using plausible alternative cut points. Cross-city comparisons should be attempted only after measurement boundaries, indicator definitions, score orientation, and cut points have been harmonized. Otherwise, outputs should be interpreted as within-city diagnostic profiles.

**Table 4:** Maturity bands used in the method.

Score range	Maturity band	Interpretation
< 2.00	Initial	Weak institutionalization or low operational reliability.
2.00–2.99	Developing	Emerging capability with partial stabilization.
3.00–3.99	Established	Operationally stable with evidence of consolidation.
≥ 4.00	Optimized	Advanced and institutionalized capability.

Step 5 **Executing the diagnostic rule engine**

The diagnostic rule engine operates on ordered time-series component scores alongside the composite and component maturity bands established in the previous step. A set of transparent rule gates is applied to observed score patterns, standardizing the initial interpretation and rendering the decision path fully auditable: every alert generated can be traced back through the chain from raw score to maturity band to rule trigger to recommended response direction. It bears emphasis that these rules do not replace expert judgment; they structure and discipline the first pass of interpretation so that analysts can direct their expertise toward cases that genuinely warrant it.

### Context adaptation of diagnostic rules

Diagnostic rules should be adapted through declared parameters rather than rewritten ad hoc. A local configuration may specify the minimum number of consecutive intervals, the volatility tolerance, priority weights, and the minimum evidence-confidence level required for a strong recommendation. The invariant core comprises explicit rule syntax, provenance and confidence tagging, a versioned configuration, and preservation of the score-to-band-to-alert-to-action trace. Every changed parameter should be reported with the results; when comparability is uncertain, analysts should run both the default and calibrated configurations and disclose any change in triggered alerts, (Table 5).

**Table 5:** Rule engine used for diagnosis.

Rule	Trigger condition	Diagnostic meaning	Recommended response
Impact Gate	Overall maturity remains Developing while Impact falls below 2.00 in any period.	The program-to-impact chain is fragile or temporarily broken.	Clarify impact definitions, audit the output-to-impact link, and strengthen evaluation routines.
Output Decline Gate	Output declines across at least two consecutive intervals.	Delivery consistency, implementation discipline, or reporting cadence is weakening.	Harmonize operational definitions, confirm delivery owners, and enforce reporting cadence.
Outcome Stabilization Gate	Outcome is moderate or relatively strong while Impact remains weak or volatile.	Intermediate achievements are visible but are not being converted into durable effects.	Create bridging KPIs that connect output, outcome, and impact measures.
Quick Win Institutionalization Gate	Quick Win reaches Established in one period and falls below that band in another.	Short-term gains exist but have not been embedded in routines.	Assign ownership, formalize SOPs, secure budget anchors, and monitor recurrence.

**Table 6:** Circularity activation tiers.

Tier	Minimum data requirement	Analytics activated	Recommendation focus
Tier 1: Minimum viable assessment	Aggregated scorecards, program records, and basic metadata.	Component scoring, maturity mapping, rule alerts, and proxy-based circularity readiness interpretation.	Governance foundations, minimum KPI set, ownership, and reporting cadence.
Tier 2: Intermediate integration	Periodic sectoral data from waste, water, energy, mobility, or service portals.	Expanded trend analysis, partial circularity computation, and confidence-adjusted alerts.	Cross-sector integration, KPI refinement, and circularity monitoring.
Tier 3: Advanced or real-time assessment	Integrated platforms, APIs, interoperable data services, and optional sensors.	Comprehensive circularity metrics, advanced diagnostics, scenario support, and continuous monitoring.	Optimization, coordination efficiency, and continuous improvement.

Step 6 **Ranking alerts and generating the policy-facing decision package**

With a list of triggered alerts in hand, the final analytical step involves ranking them according to three criteria: severity, persistence, and systemic influence. Severity is treated as high when a component descends to the Initial maturity band or when its condition undermines the credibility of the overall maturity claim. Persistence is considered high when the same pattern recurs across multiple reporting periods without resolution. Systemic influence is elevated when a weakness in one component has the potential to degrade adjacent parts of the performance chain — for instance, when deteriorating Output scores compress the validity of later Outcome and Impact readings. The output of this step is a structured decision package comprising an executive scorecard, a component maturity map, an alert panel, a ranked set of recommendations, and a 12–24–36 month policy roadmap.

### Extension module. activating circularity indicators according to local data maturity

This extension is configured after the six core stages and addresses the integration of circular economy measurement into the broader assessment. Inputs include the minimum scorecard alongside any sectoral circularity data that may be available from waste management, water systems, energy networks, mobility infrastructure, procurement records, or related sources. The method does not require comprehensive circularity data to proceed; rather, analysts select the activation tier that corresponds to their current data maturity. Where item-level or sector-level data are absent, the appropriate starting point lies in readiness assessment and governance-oriented actions. As data become more granular and interoperable over time, the indicator set can be expanded progressively. The output is a staged circularity measurement pathway calibrated to what the evidence can credibly support at each point in the city's development trajectory.

### Demonstration dataset and expected outputs

The demonstration draws on a three-year scorecard comprising five visible components. Rather than recalculating the composite from scratch, the officially reported composite is retained as the primary annual reference point, reflecting the fact that the original scorecard does not reduce neatly to the arithmetic mean of its visible parts. The arithmetic mean is computed separately and used strictly as a sensitivity check — a way of gauging how closely the official figure tracks a simple average of observable components, rather than as a replacement for it, (Table 7).

**Table 7:** Demonstration dataset.

Year	Baseline	Output	Outcome	Impact	Quick Win	Reported composite	Overall maturity
2020	2.69	2.51	3.04	2.60	3.47	2.81	Developing
2021	2.84	2.40	2.78	1.69	2.64	2.45	Developing
2022	2.85	2.07	2.92	2.75	3.48	2.83	Developing

The expected interpretation that emerges from this dataset is instructive precisely because it resists reduction to a single narrative. Overall maturity remains within the Developing band across all three years, yet component trajectories tell a more differentiated story. Output declines in each successive interval, suggesting a persistent structural weakness rather than a temporary fluctuation. Impact drops into the Initial band in 2021 before recovering in the following period, indicating a transient but significant deterioration that the composite score absorbs without surfacing explicitly. Quick Win, meanwhile, oscillates around the Established threshold, reflecting ongoing instability rather than consolidated progress.

### Pseudocode implementation

Input: annual scorecard D with year, component scores, and reported composite score

For each record in D:

validate required fields and numerical range

retain reported composite score if available

compute arithmetic mean as a sensitivity check

assign maturity band to the composite and to each component

For the ordered time series:

trigger Impact Gate if overall maturity is Developing and any Impact < 2.00

trigger Output Decline Gate if Output declines across consecutive periods

trigger Outcome Stabilization Gate if Outcome is moderate or strong while Impact is volatile

trigger Quick Win Institutionalization Gate if Quick Win moves between Established and lower bands

Rank triggered alerts by severity, persistence, and systemic influence

Generate: scorecard, maturity map, alert panel, priorities, and roadmap

Output: traceable recommendation set with evidence and suggested implementation horizon

## Method validation

Validation here is analytical, not municipal or external. The goal was to check whether the method produces reproducible, traceable, and policy-relevant outputs under the constrained data conditions it was designed for, not to claim full deployment across a real city administration. Accordingly, the tests establish implementation correctness and internal consistency only; they do not estimate predictive performance, causal validity, or cross-city generalizability.

Five analytical checks were conducted: a demonstration across all six core stages, a sensitivity analysis comparing reported composites with arithmetic means, rule-trigger verification against component trends, a comparison with a conventional scorecard reading, and a structured portability walkthrough across the three data-maturity tiers. Together, these checks verify deterministic rule execution, traceability, arithmetic stability, and architectural configurability within the demonstration. They do not establish external validity, which requires independent multi-municipality application, (Table 8).

**Table 8:** Validation tests and findings.

Validation test	Procedure	Finding
Demonstration	Run the six stages on the 2020–2022 scorecard.	The method produced a maturity map, alert panel, ranked priorities, and staged roadmap.
Sensitivity analysis	Compare the reported composite with the arithmetic mean of the visible components.	All years remain Developing under both views: 2020 = 2.81 reported versus 2.86 mean; 2021 = 2.45 versus 2.47; 2022 = 2.83 versus 2.81.
Rule-trigger verification	Apply the four diagnostic gates to component trends.	Impact Gate, Output Decline Gate, and Quick Win Institutionalization Gate are triggered; Outcome Stabilization Gate is partially triggered.
Comparison with conventional reading	Compare a composite-score narrative with the MA-SCC-DSS interpretation.	The conventional reading shows decline and recovery; the DSS localizes Output decline, Impact volatility, and weak Quick Win institutionalization.
Structured portability walkthrough	Hold the six-layer core constant while applying the input and analytics configurations specified for Tiers 1–3 in Table 6.	All tiers preserve provenance, maturity mapping, and rule-to-action logging; higher tiers progressively add indicators and analytics. This demonstrates architectural configurability, not empirical municipal validity.

### Portability boundary and external-validation protocol

The portability walkthrough shows that the workflow can be configured for aggregated, sectoral, or integrated data, but it does not show that default thresholds or rule parameters transfer unchanged across municipalities. External validation should therefore sample municipalities across all three activation tiers and heterogeneous administrative contexts; preregister the invariant core and context-specific parameters; and assess data completeness, inter-analyst agreement in triggered alerts, concordance with local expert priorities, time to diagnosis, and stability under threshold perturbation. Until such testing is completed, claims should remain limited to reproducibility, traceability, and analytical utility in the illustrative dataset.

### Policy-facing output generated by the demonstration

The method yields four ranked priorities from the demonstration dataset, each grounded in a specific pattern identified through the diagnostic process. The first and most urgent priority is to repair the impact-measurement chain, given that Impact descends to the Initial band in 2021 — a deterioration serious enough to undermine the credibility of the overall maturity claim. The second priority is to stabilize Output, which declines across every recorded interval and therefore represents a persistent structural weakness rather than an isolated anomaly. Third, bridging KPIs should be developed to connect Output, Outcome, and Impact as an integrated performance chain, rather than treating them as independent scorecard fields that happen to coexist. Fourth, Quick Win gains should be institutionalized through clearly assigned ownership, documented standard operating procedures, and dedicated budget anchors to prevent the oscillation observed around the Established threshold from continuing. Once these structural concerns are addressed, circularity indicators can be progressively activated in data-ready sectors, beginning with waste, water, energy, and mobility, (Table 9, Table 10).

**Table 9:** Staged roadmap generated by the method.

Time horizon	Main objective	Illustrative actions
0–12 months	Restore governance credibility.	Standardize impact indicators; enforce reporting cadence; confirm KPI ownership; document data provenance; define a minimum viable KPI set.
12–24 months	Improve integration and analytical breadth.	Align data dictionaries; connect sectoral data; refine confidence tags; activate partial circularity metrics for data-ready sectors.
24–36 months	Consolidate and optimize.	Expand sectoral coverage; deepen circularity analytics; introduce scenario-oriented planning and continuous feedback.

**Table 10:** Rule execution results.

Rule	Status	Evidence	Priority
Impact Gate	Triggered	Impact = 1.69 in 2021 while overall maturity remains Developing.	Very high
Output Decline Gate	Triggered	Output declines from 2.51 to 2.40 to 2.07.	High
Outcome Stabilization Gate	Partially triggered	Outcome remains moderate while Impact is volatile.	Medium
Quick Win Institutionalization Gate	Triggered	Quick Win moves from Established to Developing and back to Established.	Medium

## Sensitivity check

Table 11.

**Table 11:** Sensitivity analysis.

Year	Reported composite	Arithmetic mean	Maturity using reported composite	Maturity using arithmetic mean
2020	2.81	2.86	Developing	Developing
2021	2.45	2.47	Developing	Developing
2022	2.83	2.81	Developing	Developing

The sensitivity check serves a specific and limited purpose: it tests whether the maturity conclusion would change if a transparent alternative aggregation were substituted for the reported composite. It does not change. Under both the officially reported composite and the arithmetic mean, all three years remain classified within the Developing band. This consistency demonstrates arithmetic stability for this illustrative dataset: the maturity label does not depend on choosing one of these two aggregations. It does not establish robustness across municipalities, longer time series, or alternative cut points; those require the calibration and external-validation procedures described above.

### Comparison with a conventional scorecard reading

Table 12.

**Table 12:** Conventional scorecard reading versus DSS-based interpretation.

Aspect	Conventional scorecard reading	DSS-based interpretation
Main focus	Composite trend.	Component maturity, rule triggers, and staged action.
Main conclusion	Developing throughout; decline in 2021; recovery in 2022.	Stable overall maturity hides Output decline, Impact volatility, and weak institutionalization.
Bottleneck localization	Limited.	Strong, especially for Output and Impact.
Actionability	Low to moderate.	High: alerts, ranked recommendations, and roadmap.
Traceability	Limited.	Visible chain from score to maturity band to rule trigger to response.

**Fig. 1:**
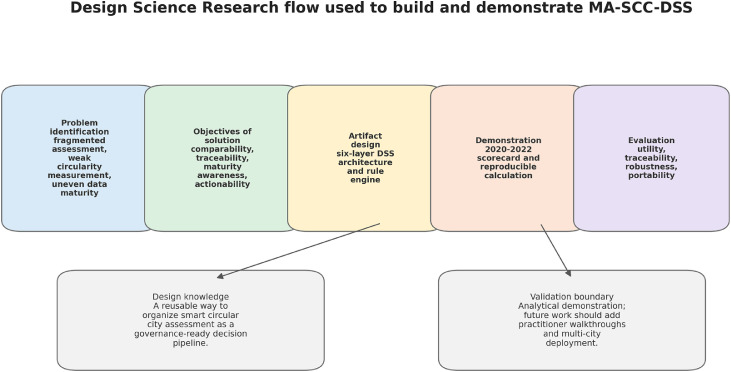
Design science research flow used to build and demonstrate the MA-SCC-DSS method.

**Fig. 2:**
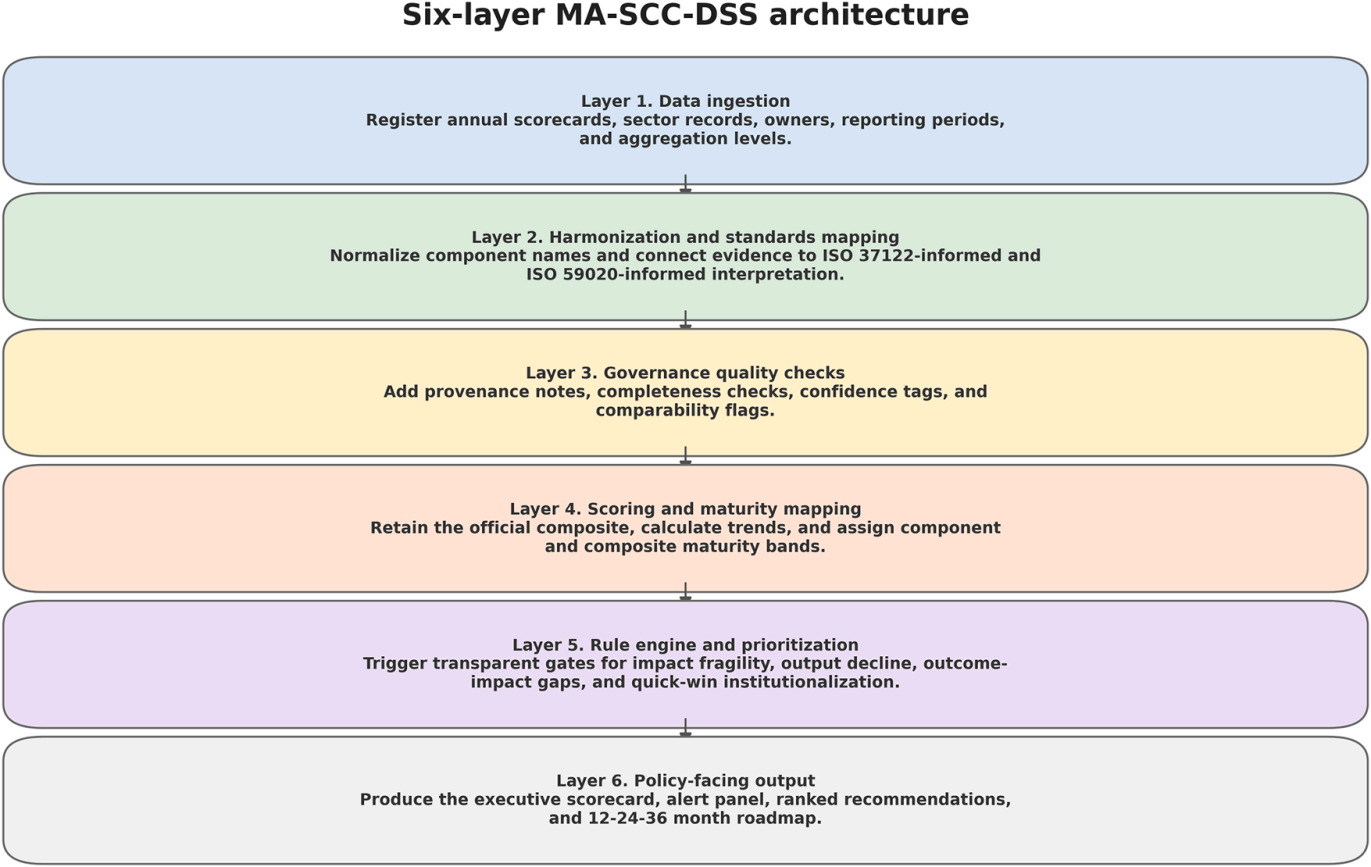
Six-layer architecture integrating data ingestion, standards-informed harmonization, governance quality control, scoring, rule diagnosis, and policy-facing output.

**Fig. 3:**
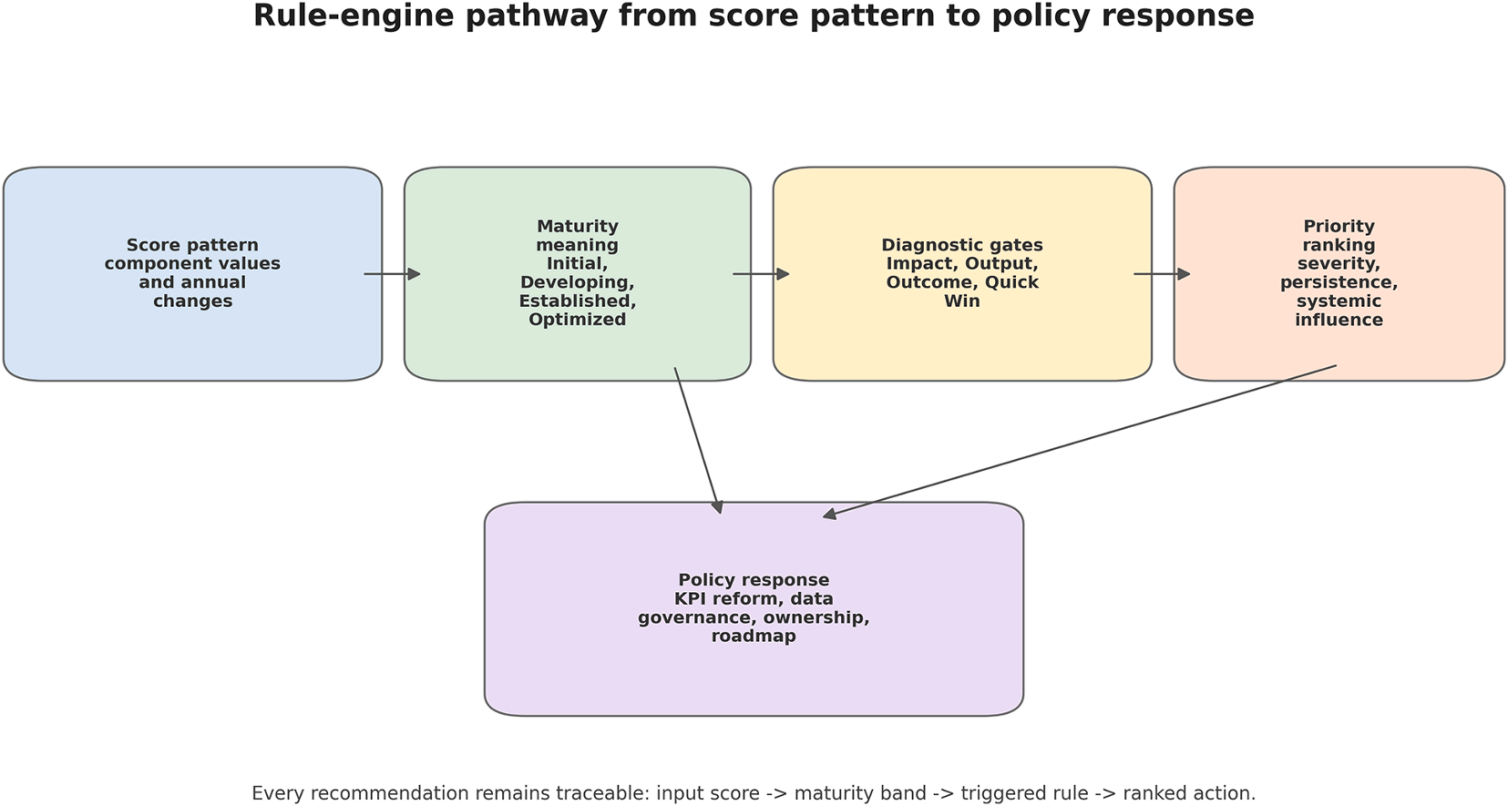
Rule-engine pathway linking score conditions, maturity interpretation, diagnostic alerts, and policy responses.

**Fig. 4:**
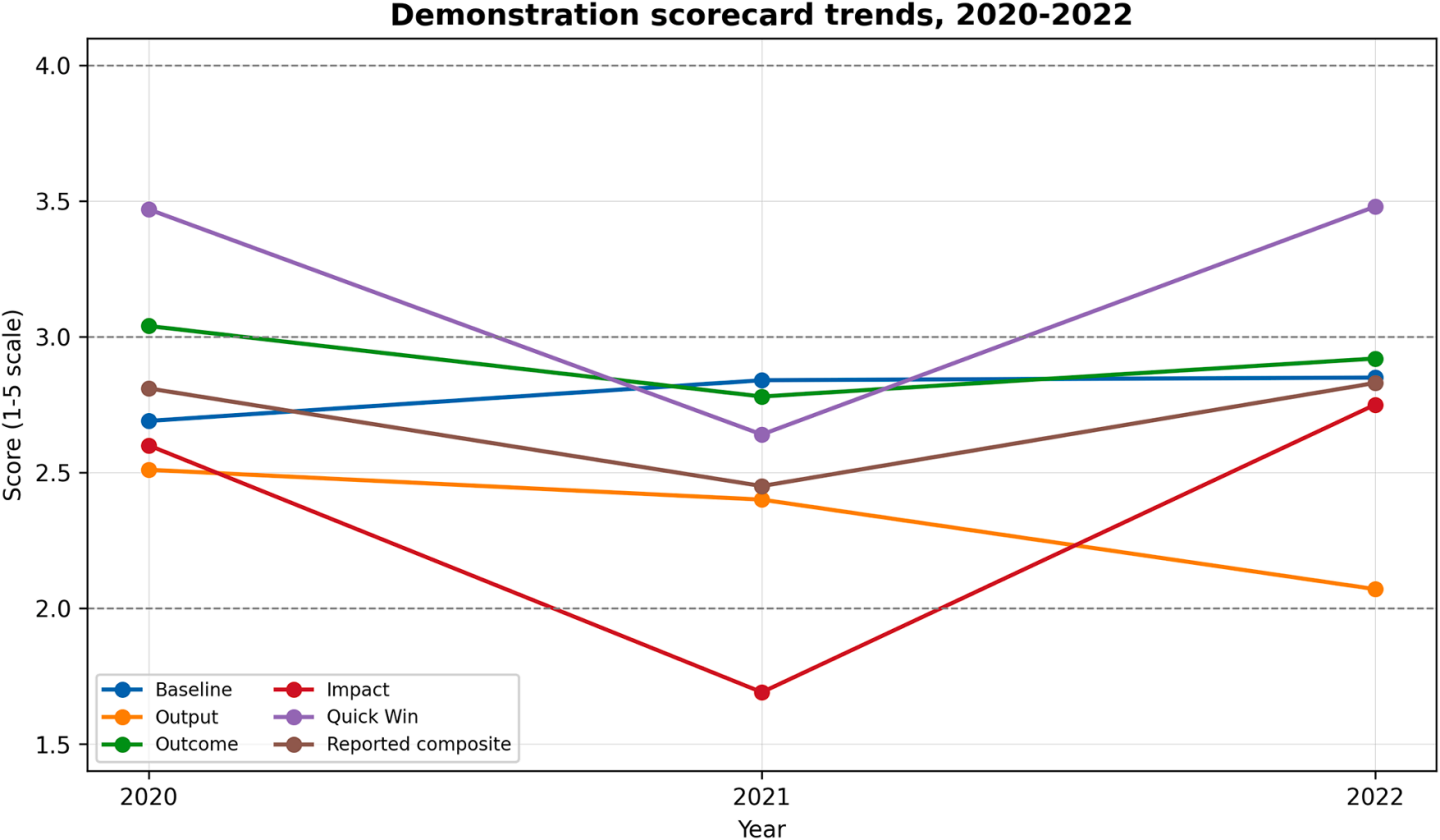
Demonstration trend profile for the 2020–2022 scorecard, showing stable overall maturity but divergent component-level trajectories.

**Fig. 5:**
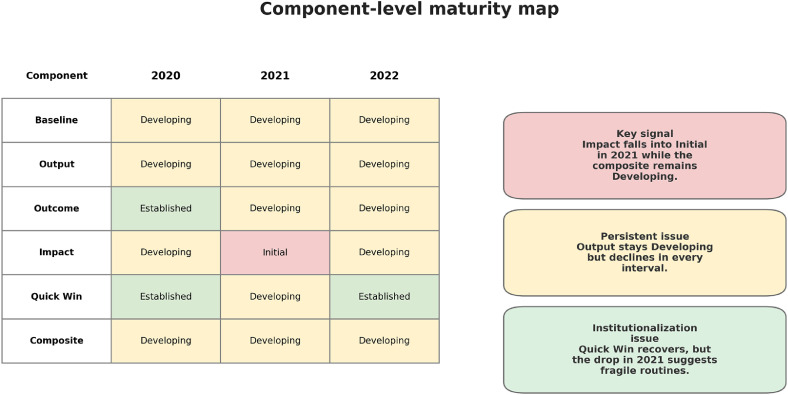
Component-level maturity map demonstration dataset.

The intended efficiency gain of MA-SCC-DSS over conventional practice is practical rather than computational. By converting a composite scorecard into a transparent chain of evidence, maturity interpretation, diagnostic rule application, and recommended action, the method is designed to reduce interpretive ambiguity. Analysts working independently from the same input data and frozen configuration should arrive at the same first-pass diagnosis and be able to explain, step by step, why each recommendation was generated. This auditability distinguishes the method from approaches that produce defensible-looking outputs without a traceable reasoning path; its actual efficiency should be measured in future field deployments (Fig. 1, Fig. 2, Fig. 3, Fig. 4, Fig. 5).

### Limitations

• The demonstration relies on aggregated scorecard components, not full sector-level circularity data. The method localizes bottlenecks; it cannot yet explain every sector-specific driver behind them.
• The circularity layer is standards-informed and staged. ISO 59,020 provides the logic, but comprehensive resource-flow accounting requires more granular data than most cities currently hold.
• Three annual observations are sufficient for a worked demonstration of rule execution, but they do not support conclusions about long-term urban transformation, threshold stability, or transferability. Broader temporal coverage and independent municipal datasets are required.
• The rule engine was built with explainability and governance utility as its primary goals. It is designed to support transparent, first-pass diagnosis that practitioners can follow and interrogate — not to establish causal inference or replace deeper analytical judgment.
• Formal ISO compliance should not be claimed on the basis of this method alone. Such claims are only appropriate where local indicators have been mapped at the item level to the relevant ISO requirements and are supported by documented, verifiable data sources.
• Future validation should extend beyond the single illustrative dataset through preregistered, multi-municipality testing across the three data-maturity tiers. It should report implementation fidelity, inter-analyst agreement, concordance with local expert priorities, time to diagnosis, and sensitivity to context-specific thresholds.

## Ethics statements

This method article uses aggregated, non-personal urban performance scorecard data for demonstration. It does not involve human participants, animal experiments, clinical material, social media data, or personally identifiable information.

## CRediT author statement

Novi Dian Nathasia: Conceptualization, Methodology, Data curation, Formal analysis, Writing - original draft, Visualization. Widowati: Supervision, Methodology, Validation, Writing - review and editing. Aris Puji Widodo: Supervision, Methodology, Validation, Writing - review and editing.

## Funding

This research did not receive any specific grant from funding agencies in the public, commercial, or not-for-profit sectors.

## Related research article

None.

## For a published article

None.

## Declaration of competing interest

The authors declare that they have no known competing financial interests or personal relationships that could have appeared to influence the work reported in this paper.

## Data Availability

A private shareable link for peer-review purposes has been provided in the paper

## Appendix Supplementary materials

Supplementary material and/or additional information Image, application 1
